# Evaluation of Trypsin-Like Enzyme Activity Using a Novel Device for Monitoring Porphyromonas gingivalis in Periodontal Pockets: A Pilot Study

**DOI:** 10.7759/cureus.83832

**Published:** 2025-05-10

**Authors:** Risako Mikami, Yasuo Takeuchi, Kazuyuki Ishihara, Yosuke Tsuchiya, Takahiko Nagai, Ryota Kobayashi, Takahiko Shiba, Takanori Iwata

**Affiliations:** 1 Department of Advanced Biomaterials, Institute of Science Tokyo, Tokyo, JPN; 2 Department of Lifetime Oral Health Care Sciences, Institute of Science Tokyo, Tokyo, JPN; 3 Department of Microbiology, Tokyo Dental College, Tokyo, JPN; 4 Department of Periodontology, Institute of Science Tokyo, Tokyo, JPN

**Keywords:** periodontal risk assessment, periodontitis, porphyromonas gingivalis, site-specific diagnosis, trypsin-like protease

## Abstract

Background/purpose: This study evaluates the potential of a novel trypsin-like protease (TLP) measurement device for site-specific periodontal risk assessment, using TLP as a marker for bacterial risk, particularly for *Porphyromonas gingivalis *(*P. gingivalis*).

Materials and methods: Thirty participants were categorized into three groups: Healthy (n = 10), Perio (untreated periodontitis, n = 9), and Perio-SPC (supportive periodontal care, n = 11). Subgingival plaque samples were collected from the deepest periodontal pocket of the maxillary anterior teeth or single-rooted premolars. TLP activity was measured using the novel device, while *P. gingivalis* counts, including those of the type II fimA strain, were quantified. Clinical parameters (including probing depth (PD), clinical attachment level, and bleeding on probing) were recorded. Statistical analyses, including Pearson’s correlation and receiver operating characteristic (ROC) curve analyses, were performed to evaluate the diagnostic performance of TLP scores.

Results: The TLP scores and *P. gingivalis *counts were significantly higher in the Perio and Perio-SPC groups than in the Healthy group. *P. gingivalis* type II fimA counts were significantly higher in the Perio group compared with the Healthy group. However, no significant difference was observed between the Healthy and Perio-SPC groups. The TLP scores strongly correlated with PD (rho = 0.80, p < 0.001), total *P. gingivalis* counts (rho = 0.92, p < 0.001), and *P. gingivalis* type II fimA counts (rho = 0.73, p < 0.001). Additionally, the ROC analysis showed high accuracy of TLP scores for detecting total *P. gingivalis* and *P. gingivalis* type II fimA counts > 10^3^/mL (area under the curve (AUC) = 0.99 and 0.93, respectively).

Conclusion: The novel TLP measurement system showed potential as a site-specific tool for periodontal risk assessment and monitoring.

## Introduction

Periodontitis is an infectious disease that destroys tooth-supporting structures and is the leading cause of tooth loss [1], strongly associated with several systemic diseases [2]. *Porphyromonas gingivalis*, an obligatory anaerobic oral bacterium and a key member of the “red complex,” is widely implicated in the pathogenesis and progression of periodontitis [3]. The current gold standard for diagnosing and monitoring periodontitis includes clinical measurements such as probing depth (PD), clinical attachment level (CAL), bleeding on probing (BOP), and radiographic evaluation. Periodontitis does not progress linearly but alternates between periods of stability and progression. Nevertheless, there are no standardized methods for monitoring disease status. Currently, clinicians rely on periodic assessments of these clinical parameters and personal experiences to predict disease progression.

With the growing demand for precision medicine in periodontal therapy, there is an increasing need for personalized diagnostic and monitoring tools that account for individual risk factors. Therefore, immunological and microbiological assessments may be useful adjuncts to complement clinical measurements. However, conventional methods for detecting periodontitis-related markers or pathogens, such as enzyme-linked immunosorbent assay (ELISA) or real-time polymerase chain reaction (PCR), require expensive equipment and significant processing time, rendering them impractical for routine clinical use.

Trypsin-like proteases (TLPs) are enzymes capable of breaking down proteins and hydrolyzing synthetic substrates such as N-benzoyl-DL-arginine-naphthylamide [4]. Many bacteria, including those associated with oral infections, produce TLP, and high TLP activity is correlated with the pathogenesis of periodontitis-associated bacteria [5]. In *P. gingivalis*, the major arginine-specific TLP activities are attributed to two proteases, RgpA and RgpB (arginine gingipains). Among various bacterial virulence factors, RgpA and RgpB play a critical role in periodontal tissue destruction by promoting fimbriae maturation, degrading host proteins, and facilitating immune evasion [6,7]. Assessing the TLP level may be a more effective approach for periodontal risk assessment and monitoring than simply detecting *P. gingivalis*, considering the variation in pathogenicity among strains.

Several diagnostic systems in which TLP is used for periodontal risk assessment have been proposed [8]. However, these systems have limitations: they cannot be used for quantitative measurements, or their use may be restricted to whole-mouth-level risk assessment. Since periodontitis progression is site-specific, developing a method that allows quantitative risk monitoring at individual sites is essential. Therefore, we aimed to develop a novel, low-cost, and time-efficient periodontitis testing system using TLP as a bacterial risk assessment marker. In addition, we included a group of patients receiving supportive periodontal care (SPC) to investigate whether TLP scores differ between untreated periodontal pockets and those with residual or recurrent inflammation following treatment. This allowed us to explore the potential clinical utility of TLP measurements in various stages of periodontal therapy.

## Materials and methods

Study design and participants

**Figure 1:**
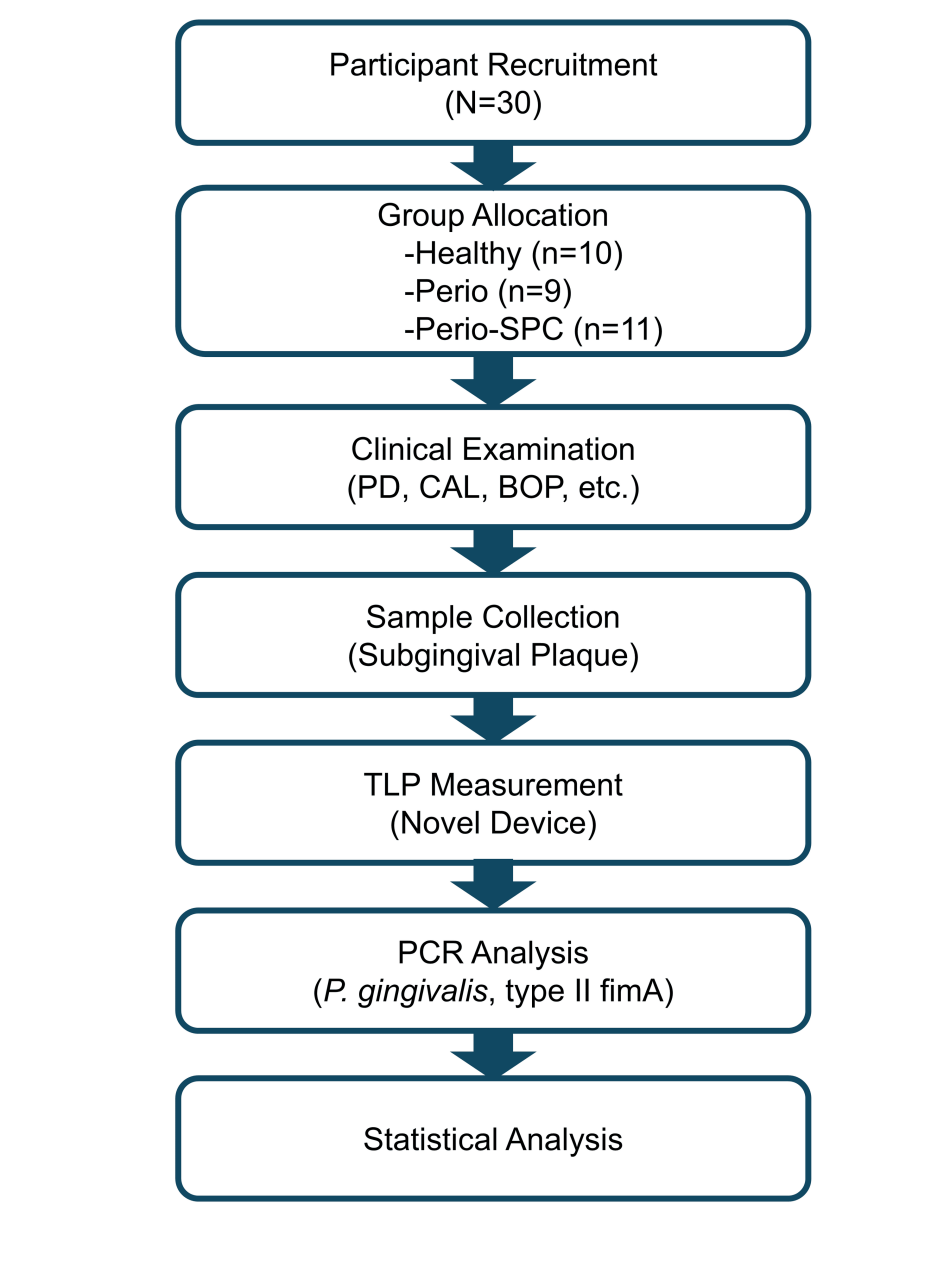
Study design overview SPC: supportive periodontal care; PD: probing depth; CAL: clinical attachment level; BOP: bleeding on probing; TLP: trypsin-like protease; PCR: polymerase chain reaction

This cross-sectional study was conducted at the Periodontal Clinic of Tokyo Medical and Dental University Hospital. The study was approved by the Dental Research Ethics Committee of Tokyo Medical and Dental University (approval number: D2020-021-02) and conducted following the Declaration of Helsinki (1975, as revised in 2013) (Figure 1).

We included (1) patients who visited the Periodontics Clinic at the dental hospital or staff from the Department of Periodontology at Tokyo Medical and Dental University; (2) individuals aged ≥20 years; (3) patients with or without periodontitis; however, for patients with periodontitis, a PD ≥ 4 mm in the maxillary anterior region or single-rooted premolars was required; and (4) patients at either the initial examination phase or SPC phase of periodontal therapy. We excluded (1) patients who smoke, (2) those with systemic diseases that could affect periodontal conditions, (3) those with acute oral symptoms, (4) pregnant or breastfeeding patients, (5) patients who had received antibiotics within the past three months, and (6) teeth with mobility grade 3.

Based on these criteria, participants were categorized into three groups: the "Healthy group" (individuals with healthy periodontal tissues), the "Perio group" (patients with untreated periodontitis), and the "Perio-SPC group" (patients undergoing SPC with either recurrence or residual periodontal pockets). All participants provided written informed consent before enrollment.

Clinical examinations

Experienced periodontists (RM, YTsu, MN, and RK) performed the clinical examinations. Periodontitis was diagnosed following the classification of the American Academy of Periodontology, categorizing patients as either healthy or having periodontitis (stages I, II, III, or IV) [9]. Among the maxillary anterior teeth or single-rooted premolars, the tooth with the deepest periodontal pocket was selected as the sample tooth. The examiners conducted the following clinical examinations at six sites on the subjected tooth using a manual probe (15 UNC Color-Coded Probe, Hu-Friedy, Chicago, IL, USA), rounding measurements to the nearest millimeter: (1) tooth mobility, (2) PD, (3) CAL, (4) BOP, (5) gingival index, and (6) plaque index.

TLP measurement

**Figure 2:**
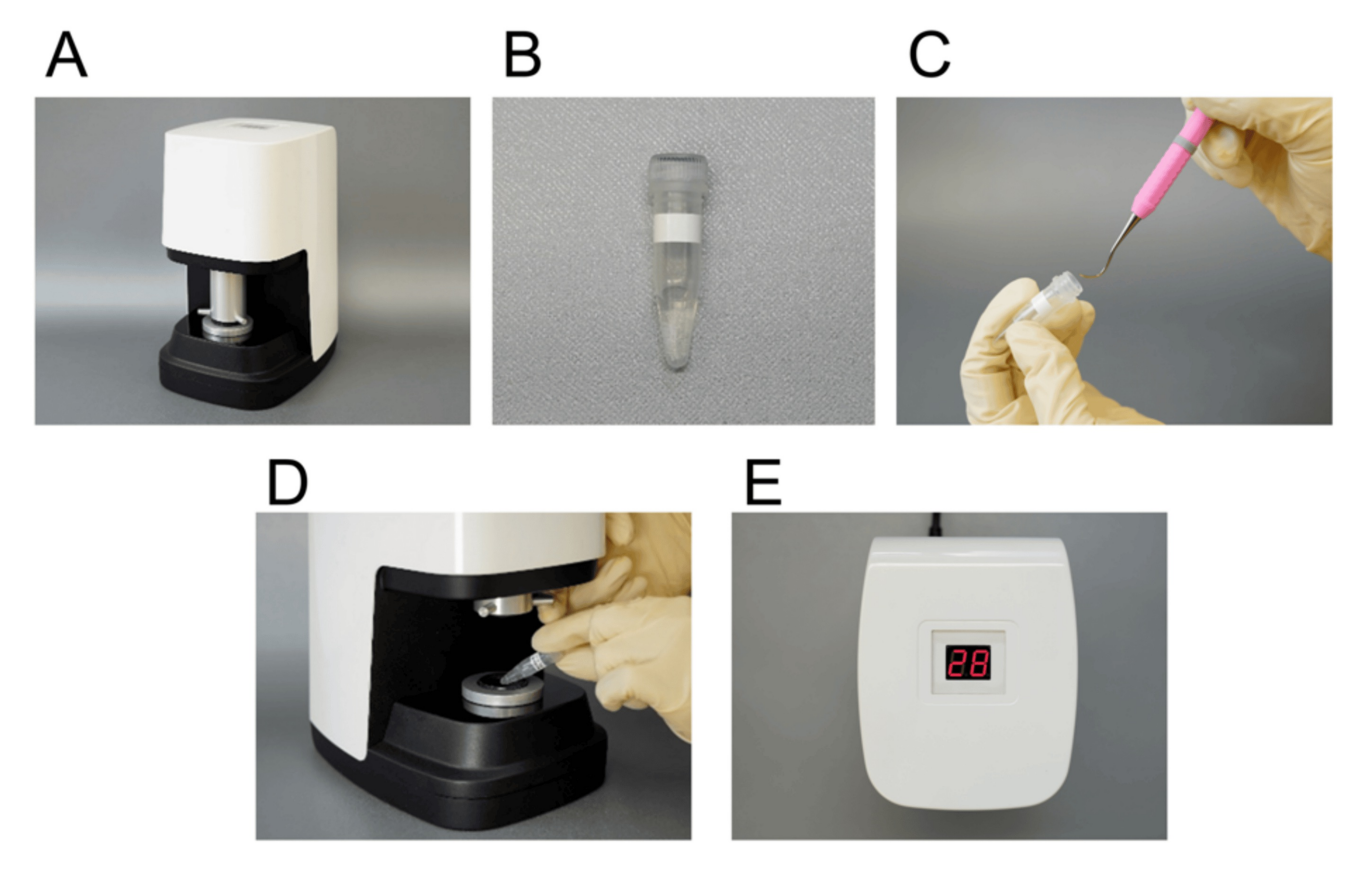
The procedure and components used for measuring trypsin-like protease (TLP) activity with the novel Dental Enzyme Checker system (A) The Dental Enzyme Checker device, (B) OEC-01 reagent, (C) subgingival plaque sample being dissolved into the OEC-01 reagent for enzymatic reaction, (D) the sample tube placed in the Dental Enzyme Checker for fluorescence-based measurement, and (E) the TLP score displayed digitally on the device screen

Subgingival plaque samples were collected from the deepest periodontal pocket of the subject tooth for TLP measurement. The sample tooth was isolated with a cotton roll, removing the supragingival plaque by swabbing with a sterilized cotton pellet and using a sterilized curette (Gracey mini curette #5, 6; Hu-Friedy, Chicago, IL, USA), inserted into the periodontal pocket, to collect the subgingival plaque attached to the root surface via two strokes. TLP activity of the sample was measured using a newly developed Dental Enzyme Checker with reagent OEC-01 (YOSHIDA Mfg. Co., Ltd., Japan). The dimensions of the device are 148 mm × 186 mm × 250 mm, and the weight is 3.0 kg. The plaque sample was mixed with OEC-01 in a commercially available tube (Screw Cap Micro Tube (Non-serrated) 1.5 mL, Sarstedt, Germany) and inserted into the Dental Enzyme Checker for measurement. OEC-01 contains methylcoumarin amide (MCA), which is linked via an amide bond to a synthetic peptide substrate (isobutyloxycarbonyl (iBoc)-Gly-Gly-Arg-MCA). This compound is non-fluorescent; however, upon enzymatic cleavage by TLPs, 7-amino-4-methylcoumarin (AMC) is released, emitting strong fluorescence (iBoc-Gly-Gly-Arg-MCA → iBoc-Gly-Gly-Arg + AMC). The fluorescence is excited at 365 nm and measured at 470 nm. This fluorescence change was measured using the Dental Enzyme Checker (Figure 2). The device was used to excite light at 365 nm and measure fluorescence at 470 nm for three minutes, and the change in the fluorescence intensity per minute was displayed as TLP activity. The enzymatic reaction was carried out at a temperature of 34°C ± 1°C, which is automatically maintained by the device during measurement. TLP activity values (TLP scores) were displayed numerically as 0-99 and could be converted to trypsin units (Try U/mL) from the standard curve of TLP score 0-99 (y = 0.9885x + 0.5358; x: TLP score, y: Try U/mL) prepared using bovine pancreatic trypsin.

Bacterial examinations

Real-time PCR was performed to quantify *P. gingivalis* cells in each sample. Genomic DNA was extracted from the samples using the DNeasy PowerSoil Kit (Qiagen, Netherlands) following the manufacturer’s recommendations. DNA samples were further amplified via a ProFlex PCR System (Thermo Fisher Scientific, Waltham, MA, USA) using a *Porphyromonas gingivalis* Genesig Advanced kit (Primer Design, UK). The DNA amplification conditions for PCR with species-specific primers for *P. gingivalis* were as follows: initial denaturation at 95°C for two minutes, followed by 50 consecutive cycles at 95°C for 10 seconds, and 60°C for 60 seconds. The number of *P. gingivalis* cells in each sample was calculated using a calibration curve. The forward and reverse primers used are listed in Table 1 [10]. Samples positive for *P. gingivalis* were additionally processed using PCR to detect type II fimA. Primers specific for type II fimA are listed in Table 2 [11]. A reaction mixture (25 µL total volume) was prepared using Ampliqon Red 2X Master Mix (Ampliqon, Odense, Denmark). The primers were used at 0.5 µM concentration, and 3 µL of the DNA template at approximately 100 ng concentration was added to the reaction mixture. The thermal cycling conditions included initial denaturation at 95°C for five minutes, followed by 35 cycles at 95°C, 58°C, and 72°C, each for 30 seconds, with a final extension at 72°C for five minutes.

**Table 1:** Primers for Porphyromonas gingivalis detection

Primer		Sequence
Porphyromonas gingivalis	Forward primer	5'-TCCTACGGGAGGCAGCAGT-3'
	Reverse primer	5'-CAACCATGCAGCACCTACATAGAA-3'
	TaqMan probe (FAM)	5'-ATGACTGATGGTGAAAACCGTCTTCCCTTC-3'GCC[TAM]

**Table 2:** Primers specific to fimA gene type II

Primer		Sequence
Type Ⅱ	fim A-F	5'-ACAACTATACTTATGACAATGG-3'
	fim A-R	5'-AACCCCGCTCCCTGTATTCCGA-5'

Statistical analysis

As this was a pilot study, no prior power calculation was performed. The sample size was determined by the feasibility and availability of eligible participants. Depending on the data distribution, continuous variables are presented as the mean ± standard deviation or as the median with interquartile ranges (25th and 75th percentiles). Categorical variables are presented as counts and percentages. *P. gingivalis* counts were log-transformed to normalize the distribution prior to analysis. Group comparisons were performed using the Mann-Whitney U test for two groups and the Kruskal-Wallis test for three groups, followed by Dunn’s post hoc pairwise comparisons with Bonferroni correction. Associations between TLP score and *P. gingivalis* count, or PD, were analyzed using Spearman’s correlation.

Receiver operating characteristic (ROC) curve analyses were performed using *P. gingivalis* counts as the reference standard to evaluate the diagnostic performance of the TLP score. The area under the curve (AUC) and Youden’s index were used to determine the predictive validity and optimal cut-off thresholds.

A p-value < 0.05 was considered statistically significant. Statistical analyses were performed using the STATA software (version 18.0; StataCorp LLC, College Station, TX, USA).

## Results

Overall, 30 sites from 30 participants were analyzed, with 10, nine, and 11 sites assigned to the Healthy, Perio, and Perio-SPC groups, respectively. The demographic data are presented in Table 3. Most participants in the Perio and Perio-SPC groups were diagnosed with stage III or IV periodontitis. The PD in each group was 1.9 ± 0.6, 5.6 ± 2.5, and 4.5 ± 1.0 mm, respectively.

**Table 3:** Characteristics of the study participants and the subject teeth PD: probing depth; CAL: clinical attachment level; BOP: bleeding on probing; SD: standard deviation; SPC: supportive periodontal care

	Healthy (n = 10)	Perio (n = 9)	Perio-SPC (n = 11)	Total (N = 30)
	Mean ± SD or n(%)	Mean ± SD or n(%)	Mean ± SD or n(%)	Mean ± SD or n(%)
Age (y)	33.3 ± 15.1	60.0 ± 15.7	68.4 ± 10.3	54.1 ± 20.3
Sex				
Male	6 (60.0%)	7 (77.8%)	5 (45.5%)	18 (60.0%)
Female	4 (40.0%)	2 (22.2%)	6 (54.5%)	12 (40.0%)
Stages of periodontitis				
Healthy	10 (100.0%)	0 (0.0%)	0 (0.0%)	10 (33.3%)
I	0 (0.0%)	0 (0.0%)	0 (0.0%)	0 (0.0%)
II	0 (0.0%)	0 (0.0%)	1 (9.1%)	1 (4.8%)
III	0 (0.0%)	6 (66.7%)	8 (72.7%)	14 (66.7%)
IV	0 (0.0%)	3 (33.3%)	2 (18.2%)	5 (23.8%)
Tooth type				
Incisor	10 (100.0%)	6 (66.7%)	6 (54.5%)	22 (73.3%)
Premolar	0 (0.0%)	3 (33.3%)	5 (45.5%)	8 (26.7%)
PD (mm)	1.9 ± 0.6	5.6 ± 2.5	4.5 ± 1.0	3.9 ± 2.1
CAL (mm)	1.9 ± 0.6	6.0 ± 3.5	5.3 ± 1.6	4.4 ± 2.7
BOP				
Negative	8 (80.0%)	0 (0.0%)	1 (9.1%)	9 (30.0%)
Positive	2 (20.0%)	9 (100.0%)	10 (90.9%)	21 (70.0%)
Gingival index				
0	8 (80.0%)	0 (0.0%)	1 (9.1%)	9 (30.0%)
1	1 (10.0%)	3 (33.3%)	7 (63.6%)	11 (36.7%)
2	1 (10.0%)	5 (55.6%)	3 (27.3%)	9 (30.0%)
3	0 (0.0%)	1 (11.1%)	0 (0.0%)	1 (3.3%)
Plaque index				
0	7 (70.0%)	0 (0.0%)	2 (18.2%)	9 (30.0%)
1	3 (30.0%)	4 (44.4%)	7 (63.6%)	14 (46.7%)
2	0 (0.0%)	5 (55.6%)	2 (18.2%)	7 (23.3%)
Tooth mobility				
0	9 (90.0%)	6 (66.7%)	4 (36.4%)	19 (63.3%)
1	1 (10.0%)	1 (11.1%)	6 (54.5%)	8 (26.7%)
2	0 (0.0%)	2 (20.0%)	1 (9.1%)	3 (10.0%)

**Figure 3:**
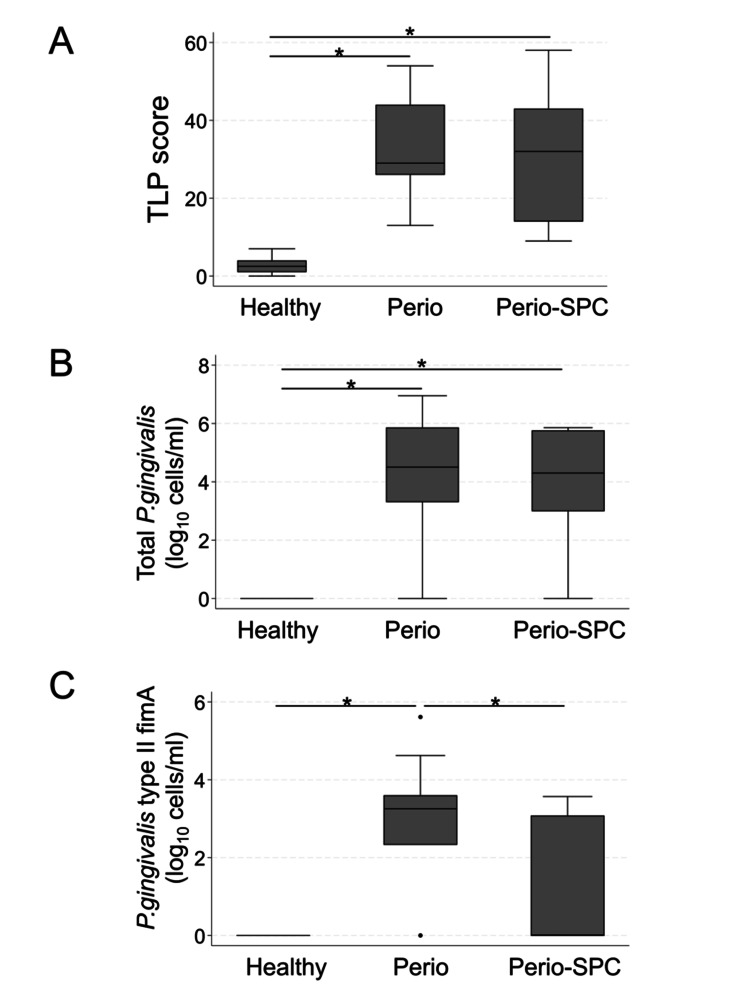
Comparison of trypsin-like protease (TLP) scores, total Porphyromonas gingivalis (P. gingivalis) counts, and P. gingivalis type II fimA counts among the Healthy, Perio, and Perio-SPC groups (A) TLP score, (B) total *P. gingivalis* counts, and (C) *P. gingivalis* type II fimA counts. Bacterial counts were log_10_-transformed after adding 1 to each value to allow for zero counts SPC: supportive periodontal care

The median TLP score of the subgingival plaques was 16 (4, 36). Table 4 shows each group’s TLP scores, total *P. gingivalis* counts, and *P. gingivalis* type II fimA counts. The TLP score and total *P. gingivalis* counts were significantly higher in the Perio and Perio-SPC groups than in the Healthy group (p < 0.001); however, no significant differences were observed between the Perio and Perio-SPC groups (Table 4, Figures 3A, 3B, p = 1.00 for TLP and p = 0.80 for total *P. gingivalis*). Regarding *P. gingivalis*, type II fimA counts were significantly lower in the Perio-SPC group than in the Perio group (Table 3, Figure 2C, p = 0.039). Additionally, the proportion of *P. gingivalis* type II fimA within the total *P. gingivalis* counts was significantly lower in the Perio-SPC group than in the other groups (Table 4, p = 0.0012).

**Table 4:** Comparison of TLP score and Porphyromonas gingivalis counts H: Kruskal-Wallis test statistic (df = 2); z: Mann-Whitney U test statistic; TLP: trypsin-like protease; SPC: supportive periodontal care ^*^p < 0.05 vs. Healthy group (Dunn's post hoc test) ^†^p < 0.05 vs. Perio group

	Healthy (n = 10) median (25%, 75%)	Perio (n = 9) median (25%, 75%)	Perio-SPC (n = 11) median (25%, 75%)	Total (N = 30) median (25%, 75%)	Test statistic
TLP score	2.5 (1, 4)	29 (26, 44)*	32 (14, 43)*	16 (4, 36)	H = 19.4, p = 0.001
Porphyromonas gingivalis					
Total (log_10_ cells/mL)	0.00 (0.00, 0.00)	4.51 (3.30, 5.86)*	4.30 (2.99, 5.76)*	3.15 (0, 4.50)	H = 14.2, p < 0.001
Type II fimA (log_10_ cells/mL)	0.00 (0.00, 0.00)	3.26 (2.33, 3.60)*	0.00 (0.00, 3.08)^†^	0.00 (0.00, 3.15)	H = 12.8, p = 0.002
Type II fimA ratio (/total, %)	-	72.3 (70.5, 77.9)	0.0 (0.0, 61.9)^†^	64.5 (0.0, 72.2)	z = 3.06, p = 0.001

**Figure 4:**
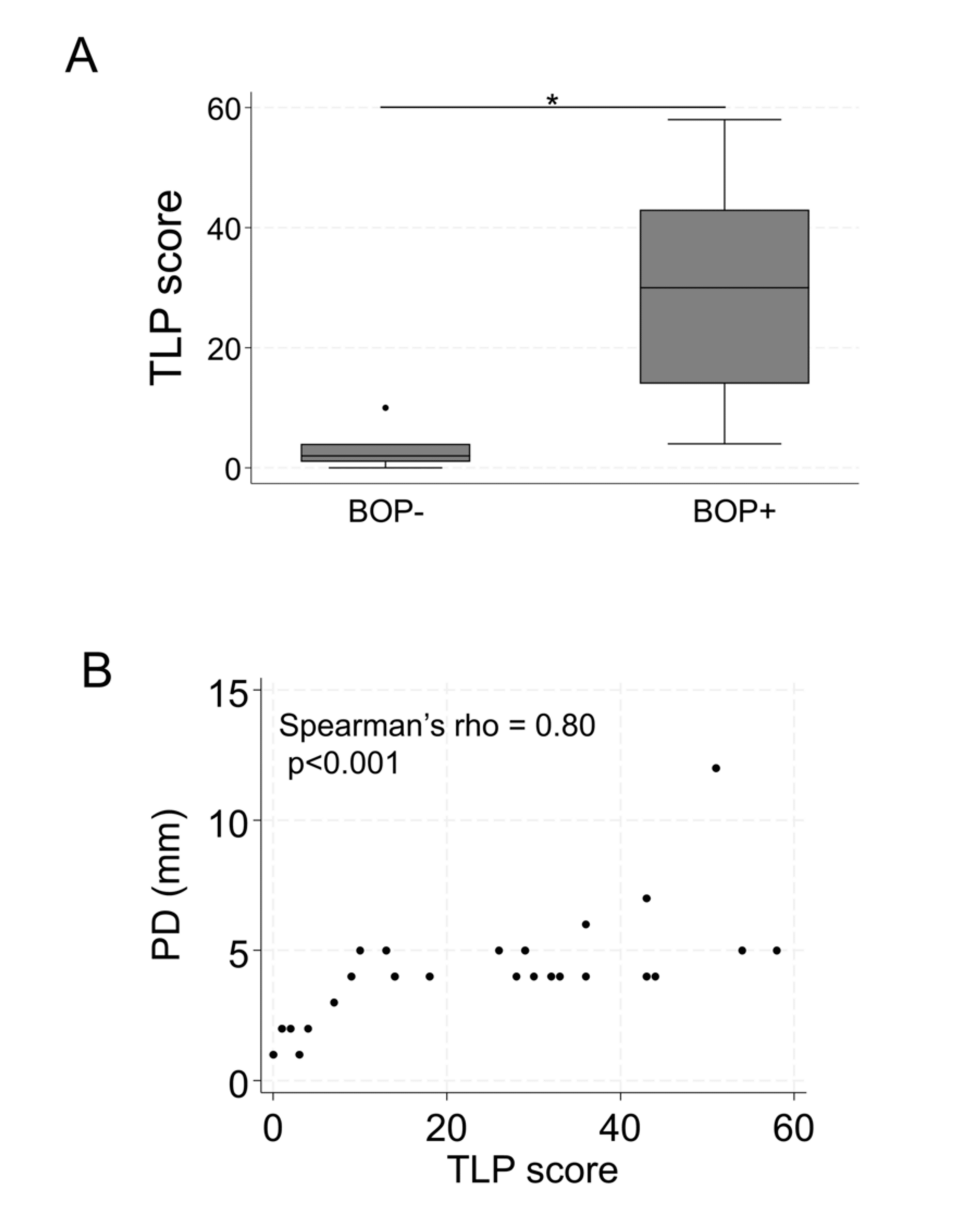
Association between clinical parameters and trypsin-like protease (TLP) score (A) Bleeding on probing (BOP) and TLP scores; (B) probing pocket depth (PD) and TLP scores

Next, we examined the relationship between periodontal parameters and TLP. The BOP-positive group had significantly higher TLP scores than the BOP-negative group (Figure 4A, p < 0.001). Furthermore, a significant positive correlation was observed between PD and TLP, with Spearman’s rho of 0.80 (Figure 4B, p < 0.001).

**Figure 5:**
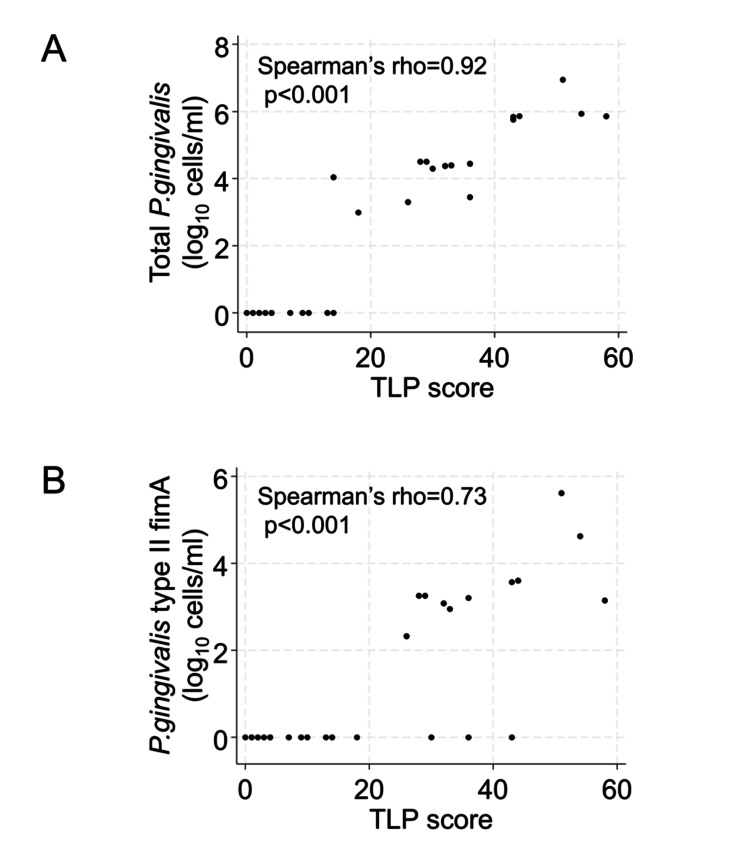
Association between trypsin-like protease (TLP) score and Porphyromonas gingivalis (P. gingivalis) counts (A) TLP score and total *P. gingivalis* score; (B) TLP score and *P. gingivalis* type II fimA counts. Bacterial counts were log_10_-transformed after adding 1 to each value to allow for zero counts

The correlation between total *P. gingivalis* count and TLP was investigated. The log_10_-transformed total *P. gingivalis* counts significantly and positively correlated with the TLP score, with Spearman’s rho of 0.92 (p < 0.001) (Figure 5A). Similarly, *P. gingivalis* type II fimA counts were significantly and positively correlated with the TLP score (Spearman’s rho = 0.73; p < 0.001; Figure 5B).

**Figure 6:**
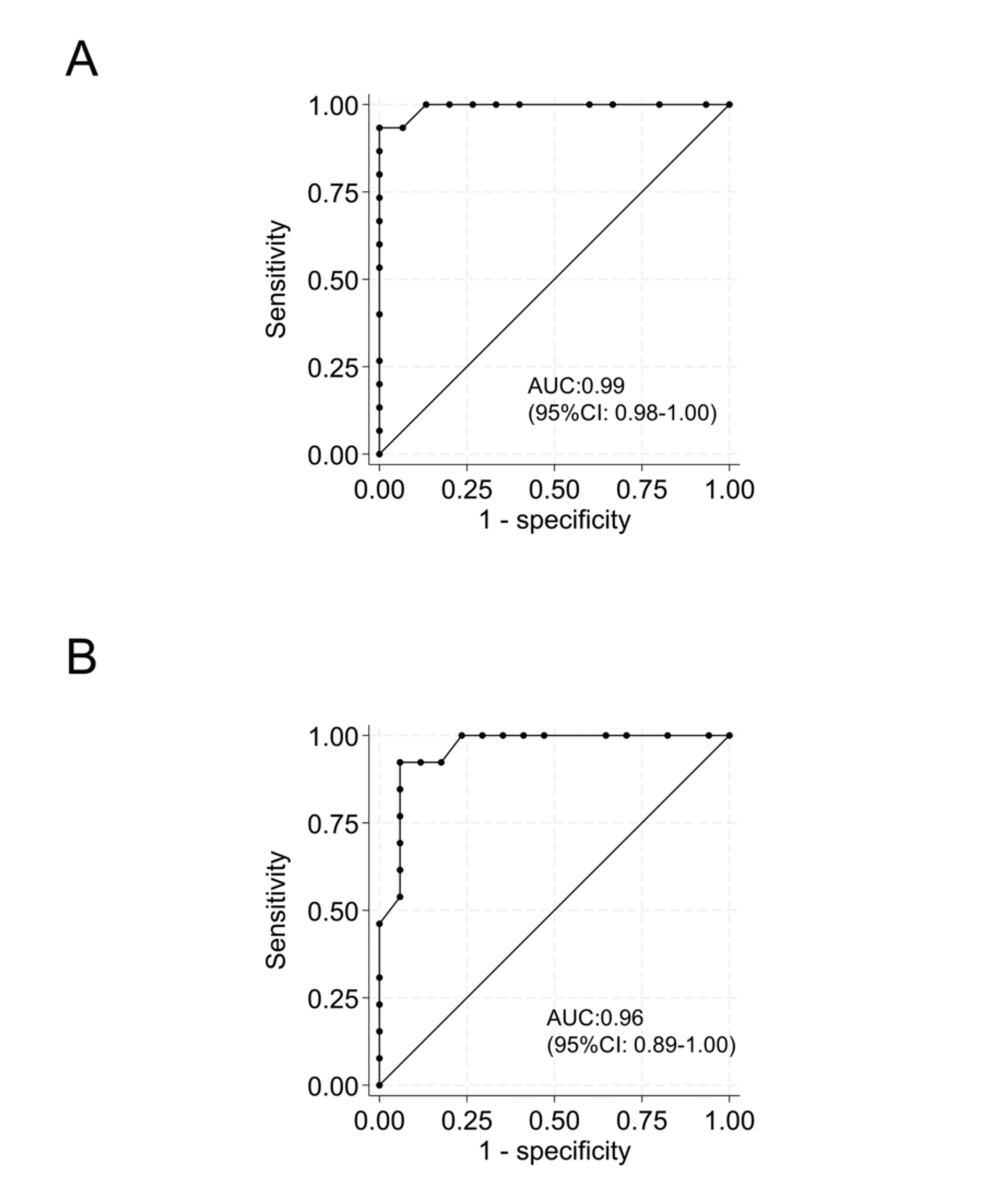
Receiver operating characteristic (ROC) curve for the trypsin-like protease (TLP) score for total Porphyromonas gingivalis (P. gingivalis) counts ROC curves evaluating the diagnostic performance of the TLP score in identifying high levels of total *P. gingivalis*. The AUC values indicate excellent diagnostic accuracy. The optimal cut-off values were determined using Youden’s index and are indicated on the curves. (A) Total *P. gingivalis* counts > 10^3^/mL; (B) total *P. gingivalis* counts > 10^4^/mL AUC: area under the curve; CI: confidence interval

The sensitivity, specificity, and correct classification of the TLP score for detecting total *P. gingivalis* counts > 10^3^/mL or >10^4^/mL in subgingival plaques are presented in Tables 5, 6. The ROC curves for the TLP scores are shown in Figure 6. The AUC of the TLP scores for detecting >10^3^/mL and >10^4^/mL of total *P. gingivalis* were 0.99 (95% confidence interval (CI): 0.98-1.00) and 0.96 (95% CI: 0.89-1.00), respectively (Figures 6A, 6B). The optimal cut-off scores for differentiating teeth with >10^3^/mL and >10^4^/mL of total *P. gingivalis* were ≥26 (sensitivity, 93.3%; specificity, 100.0%, Table 5) and ≥28 (sensitivity, 92.3%; specificity, 94.1%, Table 6), respectively.

**Table 5:** Sensitivity, specificity, and correct classification of the TLP score for Porphyromonas gingivalis counts > 10^3/mL TLP: trypsin-like protease

TLP score	Sensitivity	Specificity	Classification
≥14	100.0%	86.7%	93.3%
≥18	93.3%	93.3%	93.3%
≥26	93.3%	100.0%	96.7%
≥28	86.7%	100.0%	93.3%
≥29	80.0%	100.0%	90.0%
≥30	73.3%	100.0%	86.7%
≥32	66.7%	100.0%	83.3%

**Table 6:** Sensitivity, specificity, and correct classification of the TLP score for Porphyromonas gingivalis counts > 10^4/mL TLP: trypsin-like protease

TLP score	Sensitivity	Specificity	Classification
≥14	100.0%	76.5%	86.7%
≥18	92.3%	82.5%	86.7%
≥26	92.3%	88.2%	90.0%
≥28	92.3%	94.1%	93.3%
≥29	84.6%	94.1%	90.0%
≥30	76.9%	94.1%	86.7%
≥32	69.2%	94.1%	83.3%

**Figure 7:**
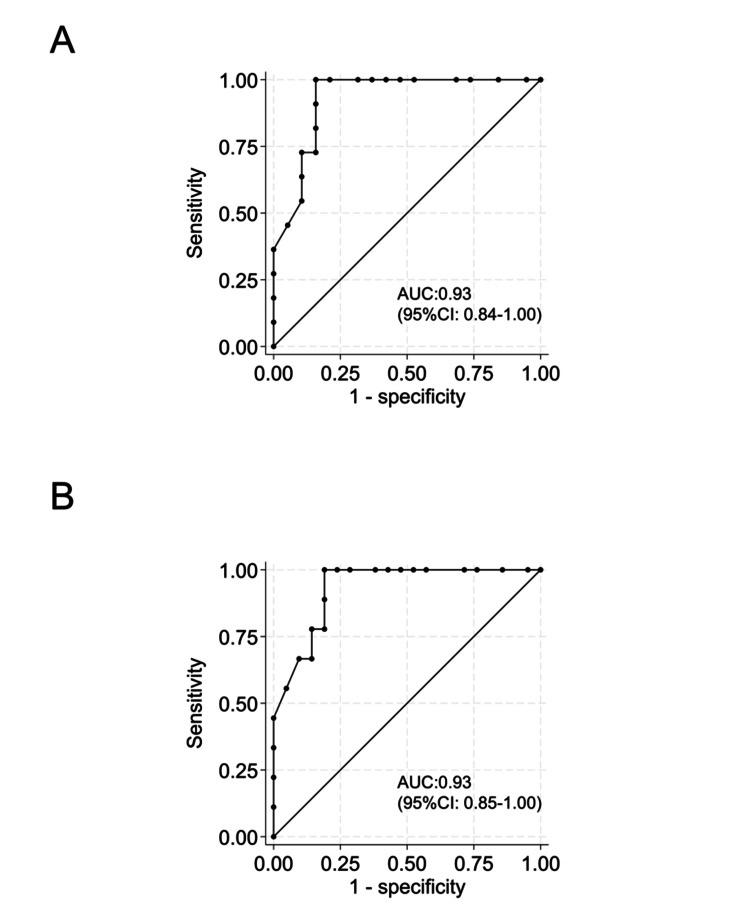
Receiver operating characteristic (ROC) curve for the trypsin-like protease (TLP) score for Porphyromonas gingivalis (P. gingivalis) type II fimA counts ROC curves evaluating the diagnostic performance of the TLP score in identifying high levels of total *P. gingivalis* type II fimA. The AUC values indicate excellent diagnostic accuracy. The optimal cut-off values were determined using Youden’s index and are indicated on the curves. (A) *P. gingivalis* type II fimA counts ≥ 10^2^/mL; (B) *P. gingivalis* type II fimA counts ≥ 10^3^/mL AUC: area under the curve; CI: confidence interval

Similarly, the sensitivity, specificity, and correct classification of the TLP score for detecting *P. gingivalis* type II fimA counts > 10^2^/mL or >10^3^/mL in subgingival plaques are presented in Tables 7, 8. The ROC curves for the TLP scores are shown in Figure 7. The AUCs of the TLP scores for detecting >10^2^/mL and >10^3^/mL of *P. gingivalis* type II fimA were 0.93 (95% CI: 0.84-1.00) and 0.93 (95% CI: 0.85-1.00), respectively (Figures 7A, 7B). The optimal cut-off scores for differentiating teeth with >10^2^/mL and >10^3^/mL of *P. gingivalis* type II fimA were ≥26 (sensitivity, 90.9%; specificity, 100.0%, Table 7) and ≥28 (sensitivity, 100.0%; specificity, 81.0%, Table 8), respectively.

**Table 7:** Sensitivity, specificity, and correct classification of the TLP score for Porphyromonas gingivalis type II fimA counts > 10^2/mL TLP: trypsin-like protease

TLP score	Sensitivity	Specificity	Classification
≥14	100.0%	68.4%	80.0%
≥18	100.0%	79.0%	86.7%
≥26	100.0%	84.2%	90.0%
≥28	90.9%	84.2%	86.7%
≥29	81.8%	84.2%	83.3%
≥30	72.7%	84.2%	80.0%
≥32	72.7%	89.5%	83.3%

**Table 8:** Sensitivity, specificity, and correct classification of the TLP score for Porphyromonas gingivalis type II fimA counts >10^3/mL TLP: trypsin-like protease

TLP score	Sensitivity	Specificity	Classification
≥14	100.0%	61.9%	73.3%
≥18	100.0%	71.4%	80.0%
≥26	100.0%	76.2%	83.3%
≥28	100.0%	81.0%	86.7%
≥29	88.9%	81.0%	83.3%
≥30	77.8%	81.0%	80.0%
≥32	77.8%	85.7%	83.3%

## Discussion

We demonstrated the potential of a newly developed TLP measurement system for site-specific periodontal risk assessment. The TLP scores showed significant correlations with the clinical parameters of periodontitis. Additionally, TLP scores were strongly correlated with bacterial counts of *P. gingivalis* and *P. gingivalis* type II fimA; *P. gingivalis* fimbriae are critical virulence factors [12], and type II fimA particularly facilitates the efficient invasion of bacteria into epithelial cells and degrades focal adhesion components, thereby compromising cellular integrity [13]. Furthermore, our comparison between untreated periodontitis sites (Perio group) and treated sites with residual inflammation (Perio-SPC group) revealed no significant difference in TLP scores, despite a reduction in *P. gingivalis* type II fimA counts in the Perio-SPC group. This finding suggests that TLP activity primarily reflects the current clinical status of individual periodontal sites, rather than the treatment history. These findings indicate that the novel TLP measurement system could be applied for site-specific periodontal risk assessment in both untreated and post-treatment contexts and may serve as a useful tool for monitoring disease activity in the future.

Periodontitis is prone to recurrence if not properly managed, and accurate diagnostic tools and consistent monitoring are essential [14]. Traditional clinical assessments remain the cornerstone of periodontal evaluation; however, laboratory-based diagnostic methods have been developed to enhance the precision of periodontitis monitoring [15]. Currently, several commercial kits for detecting periodontitis through TLP activity are available in Japan, such as BANAPERIO (Hakusui Trading Co., Ltd., Osaka, Japan) and ADCHECK^®^ (ADTEC Co., Ltd., Usa, Japan). BANAPERIO measures TLP activity produced by specific periodontal pathogens, including *P. gingivalis*, *Tannerella forsythia*, and *Treponema denticola* [4], using subgingival plaque samples. However, these systems cannot be used for quantitative measurements. Similarly, ADCHECK is a TLP activity assay kit designed for periodontitis surveillance, which allows TLP measurements using tongue swab samples, making it suitable for large-scale population screening [8]. However, because periodontitis progress is site-specific, performing site-specific monitoring is crucial for effectively tracking disease progression. Nevertheless, our novel TLP measurement system can be used to quantify TLP activity at specific sites. It may enable more efficient monitoring and risk assessment of periodontitis from a microbiological perspective without relying on expensive and time-consuming methods such as ELISA or PCR.

We also investigated the threshold values of TLP scores for detecting total *P. gingivalis* and *P. gingivalis* type II fimA counts. The TLP score showed high accuracy in distinguishing total *P. gingivalis* counts of >10^3^/mL and >10^4^/mL, as well as *P. gingivalis* type II fimA counts of >10^2^/mL and >10^3^/mL. Since there are no established thresholds for *P. gingivalis* counts [16,17], the clinical utility of TLP scores may not lie in a single-point measurement but in their potential for longitudinal monitoring. Regular TLP measurements during active periodontal therapy or SPC could enable intra-individual monitoring of *P. gingivalis* counts, providing valuable insights into periodontitis progression and recurrence risk.

In this study, the number of *P. gingivalis* type II fimA was significantly higher in untreated periodontal pockets than with the healthy sulci and the periodontal pockets in the SPC group. This suggests that the abundance of *P. gingivalis* type II fimA in periodontal pockets may differ between untreated and periodontal-treated patients. Reportedly, the detection rate of *P. gingivalis* type II fimA significantly decreased following non-surgical periodontal therapy [18], which is consistent with our study findings.

This study had some limitations. First, as a pilot study, the sample size was small. Second, we focused on *P. gingivalis*, a key pathogen within the "red complex.” Future studies should evaluate other red complex bacteria, such as *T. forsythia* and *T. denticola*, to provide an understanding of its diagnostic capabilities. Third, the thresholds used in this study were determined based on the data distribution of this study and the need to explore the diagnostic potential of the novel TLP measurement system, as there is currently no established consensus on the clinical threshold for the bacterial count of *P. gingivalis* and its impact on periodontal health. Finally, this was a cross-sectional study. Longitudinal and interventional studies are required to evaluate the system’s performance in tracking site-specific changes over time and its potential role in predicting disease progression or response to therapy.

## Conclusions

The TLP levels measured using the newly developed system were significantly correlated with periodontal clinical parameters and bacterial counts of *P. gingivalis*, suggesting that this system might be a tool for site-specific and quantitative monitoring of periodontitis.
